# QPGx‐CARES: Qatar pharmacogenetics clinical applications and research enhancement strategies

**DOI:** 10.1111/cts.13800

**Published:** 2024-05-31

**Authors:** Rania Abdel‐latif, Radja Badji, Shaban Mohammed, Wadha Al‐Muftah, Hamdi Mbarek, Dima Darwish, Duha Assaf, Daoud Al‐Badriyeh, Hazem Elewa, Nahla Afifi, Naseer Ahmad Masoodi, Amr Salah Omar, Jassim Al Suwaidi, Salha Bujassoum, Moza Al Hail, Said I. Ismail, Asma Althani

**Affiliations:** ^1^ Qatar Genome Program, Qatar Precision Health Institute Qatar Foundation Doha Qatar; ^2^ Pharmacy Department Hamad Medical Corporation Doha Qatar; ^3^ College of Pharmacy, QU Health Qatar University Doha Qatar; ^4^ Qatar Biobank for Medical Research Qatar Foundation for Education, Science, and Community Doha Qatar; ^5^ Ambulatory General Internal Medicine Hamad Medical Corporation Doha Qatar; ^6^ Cardiology and Cardiovascular Surgery Department Hamad Medical Corporation Doha Qatar; ^7^ Medical Oncology, National Center for Cancer Care and Research Department Hamad Medical Corporation Doha Qatar; ^8^ Biomedical Research Center Qatar University Doha Qatar

## Abstract

Pharmacogenetic (PGx)‐informed medication prescription is a cutting‐edge genomic application in contemporary medicine, offering the potential to overcome the conventional “trial‐and‐error” approach in drug prescription. The ability to use an individual's genetic profile to predict drug responses allows for personalized drug and dosage selection, thereby enhancing the safety and efficacy of treatments. However, despite significant scientific and clinical advancements in PGx, its integration into routine healthcare practices remains limited. To address this gap, the Qatar Genome Program (QGP) has embarked on an ambitious initiative known as QPGx‐CARES (Qatar Pharmacogenetics Clinical Applications and Research Enhancement Strategies), which aims to set a roadmap for optimizing PGx research and clinical implementation on a national scale. The goal of QPGx‐CARES initiative is to integrate PGx testing into clinical settings with the aim of improving patient health outcomes. In 2022, QGP initiated several implementation projects in various clinical settings. These projects aimed to evaluate the clinical utility of PGx testing, gather valuable insights into the effective dissemination of PGx data to healthcare professionals and patients, and identify the gaps and the challenges for wider adoption. QPGx‐CARES strategy aimed to integrate evidence‐based PGx findings into clinical practice, focusing on implementing PGx testing for cardiovascular medications, supported by robust scientific evidence. The current initiative sets a precedent for the nationwide implementation of precision medicine across diverse clinical domains.

## INTRODUCTION

Pharmacogenetics (PGx) stands at the forefront of precision medicine, offering an unprecedented opportunity to individualize pharmacotherapy.^1^ By leveraging genomic insights, PGx aims to optimize drug efficacy and minimize adverse drug reactions, ultimately enhancing patient‐centered care.^2^ The incorporation of PGx testing into clinical practice, based on established drug‐gene pairs, is now recommended by various PGx guideline groups, which is critical for maximizing patient safety and therapeutic outcomes.^3^, ^4^

In Qatar, an extensive analysis of whole‐genome sequencing data from 6000 participants in the Qatar Biobank (QBB) has revealed a high prevalence (>90%) of PGx‐relevant variants. These variants to both the efficacy and safety of several medications, all of which are covered by PGx‐based guidelines.^5^ Despite the increasing evidence associated with the clinical validity of different gene–drug pairs, the integration of PGx testing into real‐time clinical care and decision‐making has yet to be adopted widely in Qatar and faces many challenges. These challenges have mainly been associated with implementation barriers, such as limited availability of regulated clinical PGx testing, limited literacy and expertise among health professionals, technical obstacles, and the lack of integration of PGx data into electronic health records (EHR).^6^, ^7^ Furthermore, the challenge of expanding the scientific evidence base and progressing the integration of PGx into clinical settings could influence the implementation of genotype‐guided therapy at the patient's bedside.^8^

Qatar Pharmacogenetics Clinical Applications and Research Enhancement Strategies (QPGx‐CARES) is a Qatar Genome Program (QGP) initiative that aims to advance PGx in health care by standardizing its use across various healthcare settings. The initiative's key goals include establishing standardized protocols for PGx testing, interventions, interpretation, and implementation across healthcare facilities. QPGx‐CARES plans to create a comprehensive PGx database tailored to the Qatari population by integrating local data and collaborating with international databases. Collaboration among healthcare institutions, researchers, and governmental entities is significantly required to exchange insights and best practices. Additionally, educational initiatives for integrating PGx into medical curricula and providing training for healthcare professionals are a priority. These collective efforts through QPGx‐CARES aim to enhance patient care and outcomes by advancing PGx implementation in Qatar, addressing challenges, and maximizing opportunities.

As QPGx‐CARES endeavors to widely implement pharmacogenetic guidance for drug and dosage selection in clinical practice, it is crucial to establish criteria for the clinical application of PGx and assess its real‐world utility. To this end, QGP, in collaboration with Hamad Medical Corporation (HMC), the main provider of secondary and tertiary health care in Qatar, managing multiple specialized hospitals and healthcare facilities across the country under its umbrella, has spearheaded the integration of PGx testing into clinical pathways. Since 2022, QGP has initiated as a part of QGPx‐CARES several pilots targeting key cardiovascular medications such as clopidogrel, warfarin, and statins, with consistent evidence supporting the clinical validity of PGx‐guided therapy.^9^, ^10^, ^11^ The strategic direction of QPGx‐CARES focuses on the clinical implementation of PGx to bridge current research gaps and overcome challenges associated with PGx application in everyday healthcare in Qatar. Additionally, the current implementation projects aim to empower practitioners with access to PGx test results, leveraging genomic data for clinical benefit by developing clinical decision support tools (CDS), thereby enabling clinicians to prescribe the right medication at the right dose.

## QPGx‐CARES: QGP INITIATIVE TO FOSTER PGx TESTING IN QATAR

**FIGURE 1:**
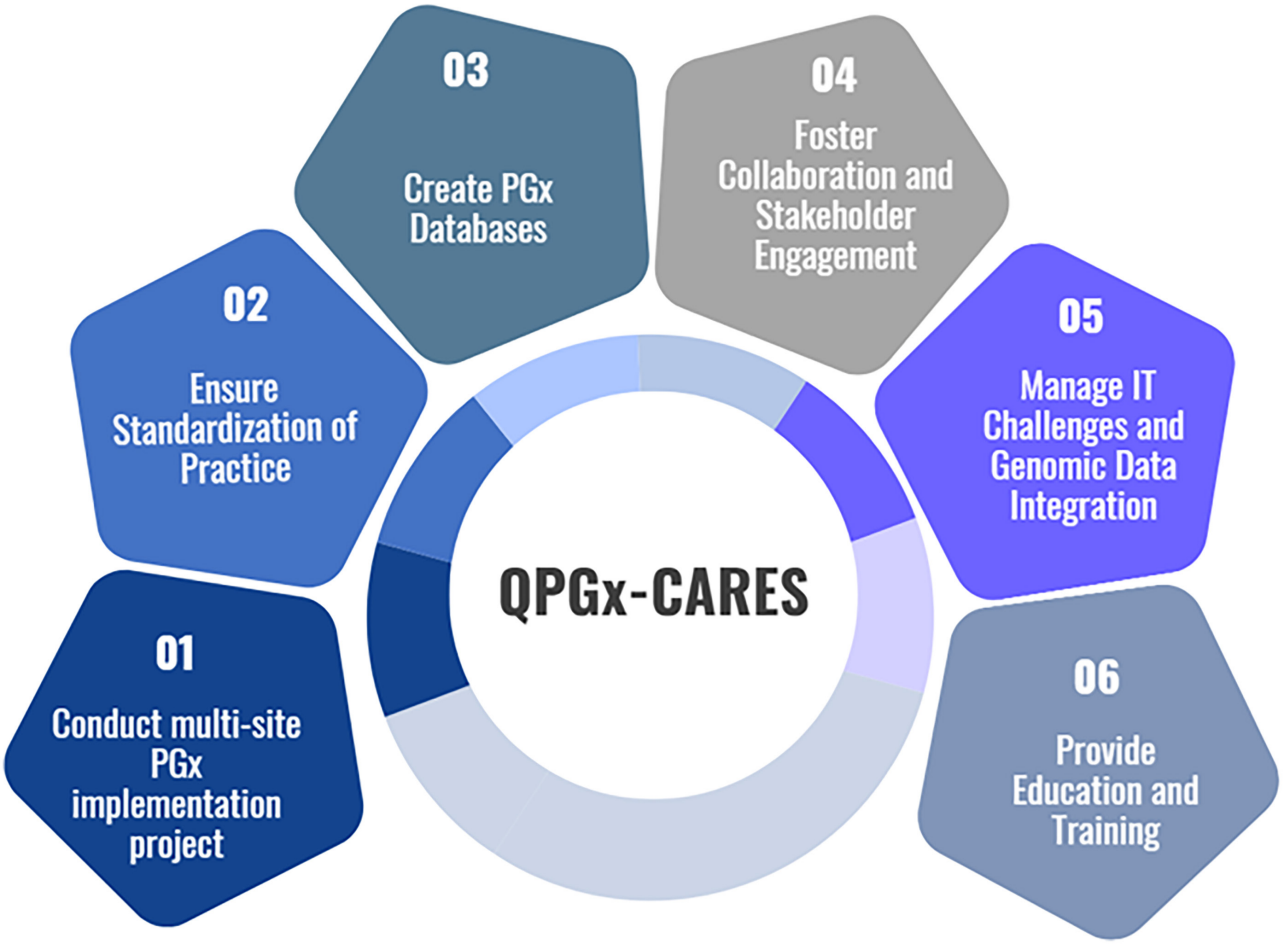
QPGx‐CARES key elements for advancing pharmacogenetic research and clinical implementation in Qatar.

PGx holds immense potential to revolutionize health care by tailoring treatments based on an individual's genetic makeup. To highlight its benefits and facilitate widespread integration, QPGx‐CARES is proposed as a coordinated multi‐site initiative across healthcare facilities in Qatar. This initiative aims to establish standardization of PGx clinical implementation in different health care settings in Qatar. Standardization of practice will ensure uniformity and efficacy of PGx clinical implementation through standardized protocols and guidelines to incorporate PGx testing and interventions into clinical practice in Qatar. Additionally, the initiative aims to create a unified approach for PGx testing, interpretation of results, and implementation of recommendations across healthcare institutions. Throughout setting standards for testing, reporting, and integration into EHR, QPGx‐CARES approach will further streamline the PGx clinical implementation process. QPGx‐CARES will establish a comprehensive PGx database specific to the Qatari population, through integrating data from QGP and QBB (Figure 1). The QPGx‐CARES database will receive regular updates and undergo curation, and measures will be taken to ensure accessibility to healthcare providers. Additionally, engaging in partnerships with international databases will enrich the diversity and depth of available PGx data. Through our initiative, investments in robust information technology (IT) infrastructure are a priority to enable the handling of large‐scale genomic data securely and seamlessly integrating it into clinical systems. QPGx‐CARES is developing standardized data formats and protocols to streamline integration into EHR and CDS. In this regard, we are collaborating with various scientists to develop effective algorithms and tools for integrating and analyzing genomic data.

QPGx‐CARES focuses on fostering collaboration among healthcare institutions, researchers, and government entities to align efforts and resources toward PGx implementation. Collaboration between institutions, clinicians, and researchers will enable shared insights and best practices derived from PGx pilot projects. To further support the healthcare professional training, QPGx‐CARES will encourage the incorporation of PGx education into medical and allied health curricula. Additionally, we will provide comprehensive training programs for healthcare professionals on PGx concepts, methodologies, interpretation, and integration into practice. Continuous education programs are currently developed through workshops and seminars in the academic institution in Qatar.

The coordinated efforts through the QPGx‐CARES initiative are pivotal in advancing PGx implementation in Qatar. Through collaboration, education, infrastructure development, and standardization, the state of Qatar can bridge gaps, overcome challenges, and leverage opportunities to facilitate the effective integration of PGx into clinical practice and, ultimately, enhance patient care and outcomes.

## STEPWISE APPROACH FOR PGx CLINICAL IMPLEMENTATION

**FIGURE 2:**
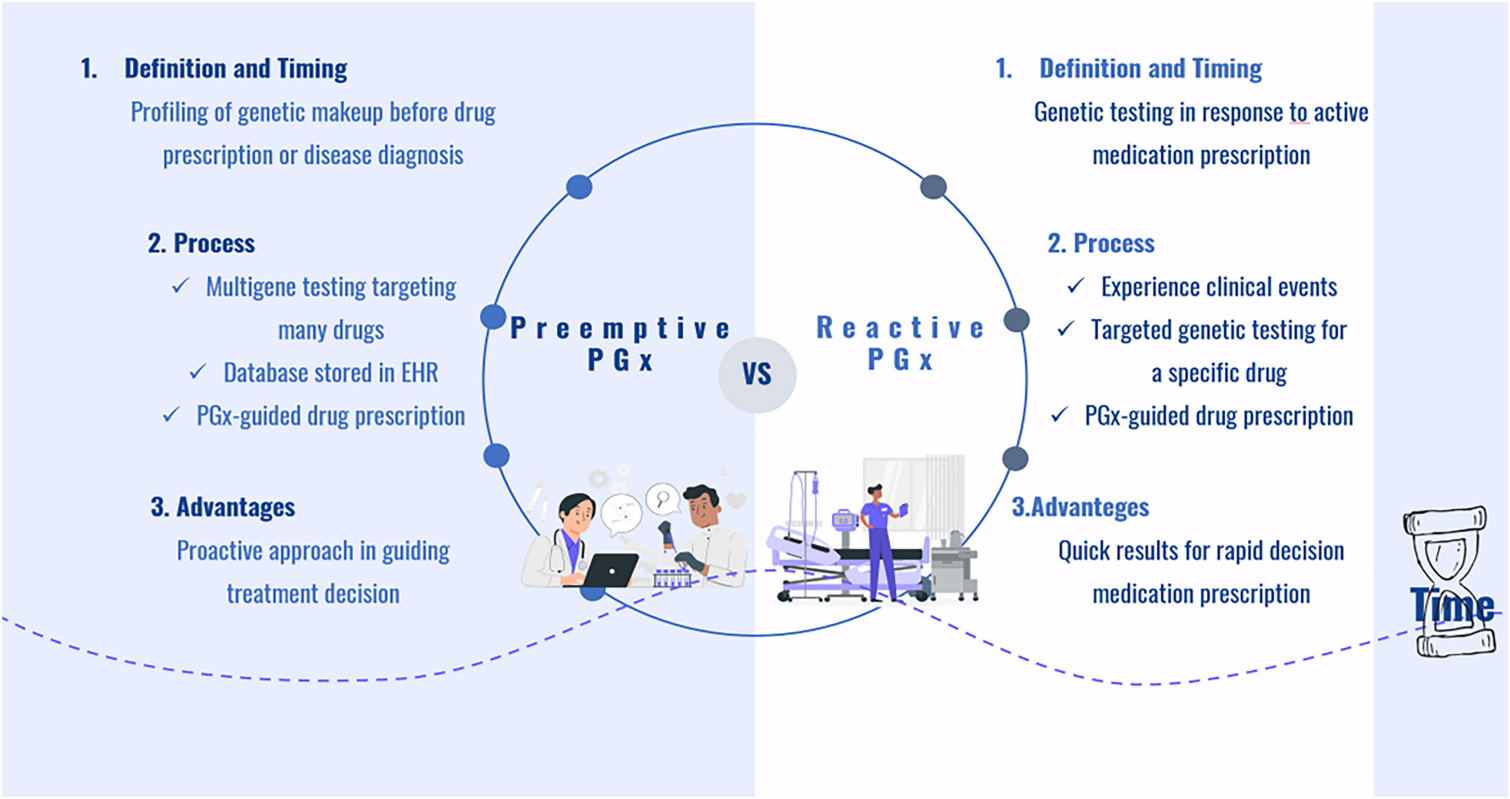
Schematic frame for preemptive and reactive PGx models.

In two modes, PGx testing can enhance clinical evaluations regarding current or future drug therapies.^12^ The first model is referred to as the point‐of‐care (POC) model or reactive model. This model reactively addresses only a few targeted gene–drug combinations and is usually prompted by prescriptions for high‐risk medications or as a consequence of emerging adverse drug reactions. The second mode is a preemptive model, which analyzes a broader range of genes to provide a more comprehensive view of an individual's drug response. The preemptive PGx model addresses potential drug therapies before a disease becomes symptomatic, and a drug with PGx information is prescribed.^13^ Integrating the results into patients' EHR can made this information accessible at the point of care (Figure 2).

QGPx‐CARES plans to provide PGx‐guided therapy for the most common diseases in the country with substantial morbidity and cost burdens, including cardiovascular, cancer, and mental health medication. For the current phase, single and multiple‐gene test assays are conducted in the cardiovascular arena, employing both reactive and preemptive models for the most prominent genes.

As part of QPGx‐CARES for PGx implementation strategy, we prioritize initiating pilot projects before embarking on widespread systemic implementation of the service. These pilot projects serve a dual purpose: They will provide insights into the challenges associated with implementation and, by addressing them, can improve the chances of successful adoption and implementation within healthcare systems. Second, they enable us to gauge the clinical utility of PGx testing and effectiveness of PGx‐guided therapy compared with routine therapy.

QPGx‐CARES initial PGx pilot implementations focus cardiovascular medicine, which have proven significance and feasibility, supported by strong evidence base showcasing their clinical relevance.^14^ To improve the applicability of PGx test results, evidence‐based guidelines, created by professional societies such as the Clinical Pharmacogenetics Implementation Consortium (CPIC) and Dutch Pharmacogenetics Working Group, are available.^15^ Several consortia have contributed to the creation of practical guidelines that translate PGx discoveries into clinical recommendations. These recommendations encompass drug dosage adjustments and considerations for alternative medications that facilitate the adoption of personalized prescribing within healthcare settings.^16^, ^17^ In the initial phases of our implementation pilots, the genetic testing results are paired with the therapeutic guidelines obtained from the Food and Drug Administration (FDA) Table of Pharmacogenomic Biomarkers in Drug Labeling and CPIC guidelines. Notably, these tests cover gene–drug pairs such as CYP2C19 with clopidogrel, CYP2C9/VKORC1 with warfarin, and SLCO1B1/ABCG2/CYP2C9 with statins (Table 1).

**TABLE 1:** Overview of the selected gene–drug pairs, their potential consequences, and CPIC recommendations.

Medication	Class of drugs	Potential for PGx‐testing	Actionable‐tested genes	Clinical evidence	CPIC clinical recommendation	Ref
Clopidogrel	CVS/anti‐platelets drugs	Individual variability related to antiplatelet efficacy	CYP2C19	CYP2C19*2 (c; rs4244285) and CYP2C19*3 (c.636G>A; rs4986893) are linked to clopidogrel high‐on treatment platelet reactivity	For poor metabolizers, avoid clopidogrel if possible. Consider an alternative P2Y12 inhibitor at standard dose if clinically indicated and no contraindication	9
Warfarin	CVS/anti‐coagulants	Variations in warfarin dose requirements essential for achieving and maintaining INR	VKORC1 CYP2C9	CYP2C9*2 (c.430C>T; rs1799853), CYP2C9*3 (c.1075A>C; rs1057910) and VKORC1 –1639G>A linked to increase warfarin sensitivity	The clinical and genetic information used in PGx algorithm‐based warfarin dosing for dosing adjusting	10
Statins	CVS/HMG‐CoA reductase inhibitors‐Anti‐hyper‐lipidemic	Preventing SAMS experienced by subset of statin‐treated individuals	SLCO1B1 CYP2C9	SLCO1B1 c.521T>C (rs4149056), ABCG2 (c.421C>A, rs2231142) CYP2C9*2 (p.R144C; rs1799853) and CYP2C9*3 (p.I359L; rs1057910) is linked to SAMS	For poor or decreased function, consider reduced dose or alternative statins for high/moderate intensity statins according to the guideline	11

Abbreviations: CVS, cardiovascular; INR, International Normalization Ratio; SAMS, Statin Associated Muscle Symptoms.

The Minor Allele Frequency (MAF) of the actionable genotypes associated with the targeted cardiovascular medications are in different populations. Data from Genome Aggregation Database (GenomAD) are used to get the allele frequency information for various subpopulations include South Asia, East Asia, Amish, African/African American, European (both Finish and non‐Finish), Ashkenazi Jewish, and Middle Eastern populations.

**FIGURE 3:**
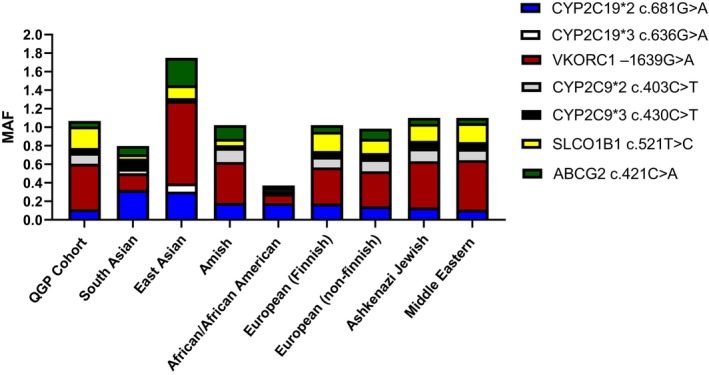
The distribution of minor allele frequency among various populations for the studied cardiovascular medications‐related actionable genotype.

The MAFs between the current QGP cohort and eight subpopulations for all examined actionable genotypes is illustrated in Figure 3. This analysis offers insights into how allele frequencies observed in the QGP cohort compare to those of other populations, providing valuable information regarding genetic diversity and potential implications for personalized medicine.

Regarding clopidogrel‐related actionable genotypes, the CYP2C19*2 actionable variant associated with clopidogrel response exhibited consistency across all ethnic groups. Conversely, the MAF of the CYP2C19*3 allele was generally low, approaching zero for most populations, except for the Eastern Asian population, where it showed a relatively high frequency of 0.088. Consistent with Amish, European, Ashkenazi Jewish, and Middle Eastern populations, the VKORC1 –1639G>A allele frequency associated with warfarin sensitivity displayed relatively high MAFs in the QGP cohort, reaching up to 0.492. This contrasts with the relatively low MAF observed in South Asian and African American populations (0.178 and 0.997, respectively).

Additionally, the CYP2C9*2 and CYP2C9*3 alleles associated with both warfarin and statin actionable responses demonstrated relatively stable MAFs across most populations. Interestingly, the MAF of SLCO1B1 c.521 T>C, linked with statin‐related myopathy in the QGP cohort, was highest among the other subpopulations, reaching up to 0.2322. Conversely, the MAF displayed for ABCG2, another variant associated with statin‐related myopathy, showed relatively low frequency across all subpopulations except for the East Asian population, where it exhibited a relatively high frequency of 0.298.

Cardiovascular medications exhibit wide interpatient variability in safety, efficacy, and dosage requirements; clopidogrel and warfarin were selected as the best candidates for the project. In the clinical setting, warfarin dosing variability is highly challenging, affecting both bleeding and thrombotic complications.^18^ Likewise, clopidogrel demonstrates significant interpatient variability in its antiplatelet effects, which can affect its effectiveness in preventing cardiovascular events.^19^ Since warfarin and clopidogrel are both considered immediate‐decision medications, POC testing is used to provide reliable genetic results in less than an hour. Having both test results and the knowledge of the patient's medication requirements allows fast interpretation of outcomes and the therapy optimization in a timely manner. For genetic interpretation and clinical recommendations for eligible patients, the CPIC guidelines are used to guide dosage and drug selection.

### Clopidogrel_CYP2C19 implementation project

The Clopidogrel_CYP2C19 implementation project is the utilizing of PGx testing to guide clopidogrel prescription, an antiplatelet agent that is crucial for managing ischemic heart disease and cerebrovascular disease. The efficacy of clopidogrel is tightly related to its metabolic activation by the CYP2C19 enzyme.^20^ However, a certain loss‐of‐function (LOF) variants in CYP2C19 gene, specifically CYP2C19*2 or CYP2C19*3, impairs this process, leading to inadequate platelet inhibition and a consequent rise in cardiovascular risk.^21^ A study published by the Qatar genome consortium has shown that CYP2C19 LOF polymorphism is prevalent among the Qatari population, occurring at a frequency of 21.7%.^5^ Patients harboring these polymorphisms have high platelet reactivity during treatment and an increased risk of adverse cardiovascular events.^21^ Recognizing the clinical significance, our project has specifically targeted patients undergoing percutaneous coronary intervention (PCI) who are prescribed clopidogrel as part of their antiplatelet therapy. Our hypothesis suggested that patients treated with clopidogrel, who are carrying CYP2C19 LOF alleles, may face an elevated risk to major adverse cardiovascular events (MACE) and stent thrombosis. This high risk has been underscored by the FDA's boxed warning regarding clopidogrel. The cautionary note advises healthcare providers to consider alternative antiplatelet therapies, such as prasugrel or ticagrelor, for individuals carrying the genetic variants.^22^

Initially, the project collaborated with a selected group of interventional cardiologists to evaluate their response to utilizing genetic information in prescribing medication for PCI patients. The Cardiac Catheterization Laboratory at HMC, serving as the primary center for such procedures, has a nationwide reach, increasing the impact of its services. As part of the cardiologists' regular clinical practice, CYP2C19 PGx tests are not requested. Therefore, we introduced onsite testing using an analytically validated POC genotyping analyzer. Adopting this POC PGx testing platform is anticipated to reduce treatment delays by providing rapid results, aligned with the urgent need for clopidogrel in PCI settings. Moreover, the project incorporates phenotyping via turbidimetric optical detection (using the VerifyNow® P2Y12 analyzer) to assess platelet function ex vivo. This dual approach of genotyping and platelet reactivity testing informs the personalization of antiplatelet therapy, adhering to CPIC guidelines. These guidelines recommend that carriers of the LOF CYP2C19 allele avoid clopidogrel in favor of alternatives like ticagrelor or prasugrel, barring contraindications, while noncarriers may proceed with standard clopidogrel dosing.^9^

**FIGURE 4:**
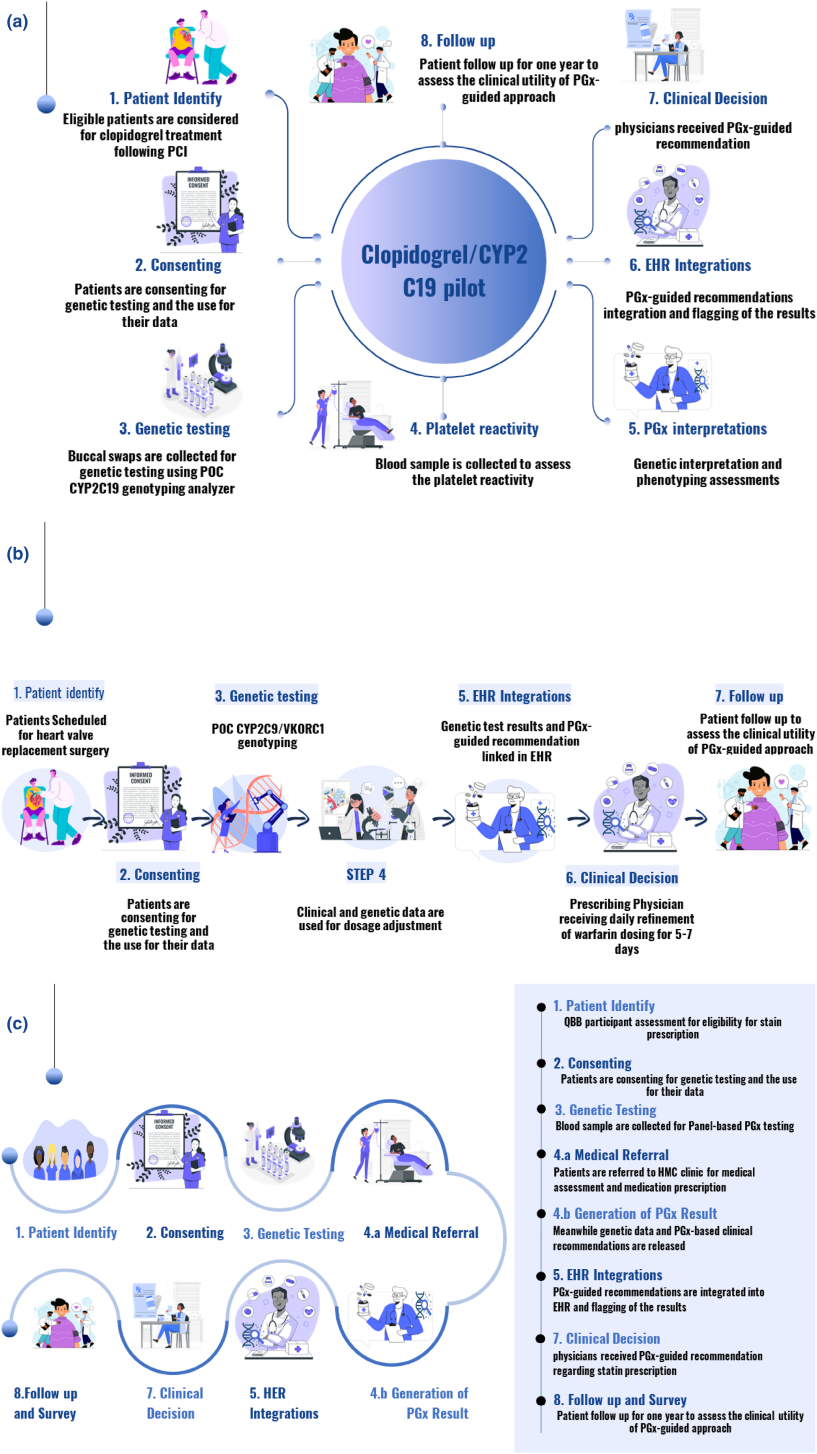
A schematic workflow of QGP/PGx pilot implementation (a) Clopidogre_CYP2C19 implementation pilot (b) Warfarin_CYP2C9/VKORC1 implementation pilot (c) Statins_SLCO1B1/ABCG2/CYP2C9 Implementation. EHR, Electronic Health Record; PCI, Percutaneous coronary intervention; PGx, Pharmacogenetics; POC, Point of Care.

The CYP2C19 genotyping is conducted as a research study. We have secured IRB approval, and patients are providing informed consent for the clinical and research use of their genetic data, which are integrated into their EHR. The ordering physician receives notification for PCI patients identified with an actionable genotype (either intermediate or poor metabolizers) who possess a corresponding PGx report in their EHR. This notification alerts the prescribing physician that the patient's CYP2C19 genotype may reduce clopidogrel efficacy, suggesting that an alternative antiplatelet therapy is advisable. Following this protocol, we also ensure regular follow‐up for up to a year to document the clinical utility of this pharmacogenomic intervention. Since the program's launch in February 2023, nearly 200 patients have had clopidogrel PGx reports added to their medical records by November 2023. The project's streamlined process is illustrated in Figure 4a.

### Warfarin_CYP2C9/VKORC1 implementation project

The second QPGx‐CARES implementation project involves a clinical implementation of PGx‐guided warfarin dosing in patients undergoing heart valve replacement (Figure 4b). In Qatar, warfarin therapy is typically initiated using empirical dosing post‐heart valve replacement surgery. The coagulation status is regularly monitored by keeping the international normalized ratio (INR) value in the target range, making the precision of the anticoagulant therapy of warfarin challenging. Warfarin is an anticoagulant therapy for the prevention and treatment of thromboembolic disorders associated with various conditions, including heart valve replacement surgery.^23^ Warfarin treatment is complicated by the significant variability in the individual response, besides its narrow therapeutic and higher drug interaction liability, which can lead to unpredicted dose requirements and, hence, increased risk of thrombosis or/and bleeding.^24^, ^25^ Genetic polymorphisms significantly influence individual warfarin dose requirements, particularly variants in the vitamin K epoxide reductase complex subunit 1 (VKORC1) gene—the target enzyme of warfarin—and the cytochrome P450 family 2 subfamily C member 9 (CYP2C9) gene, which is involved in warfarin metabolism.^26^ In addition to genetic factors, non‐genetic factors such as age, smoking status, concurrent medication use, and comorbid conditions account for roughly 50% of dose variability, with dose variations reaching up to 40% in Qatari patients.^27^ Those with the VKORC1–1639G>A variant are typically more sensitive to warfarin, while carriers of the LOF CYP2C92 and VKORC1 alleles require lower doses due to impaired metabolism.^28^, ^29^

Based on accumulating data, the FDA has updated the drug label for warfarin and recommended considering CYP2C9 and VKORC1 genotype information during the selection of warfarin dosing. Furthermore, warfarin CPIC guidelines, updated in 2017, strongly recommend prescribing warfarin dosing based on the patient's genotype.^10^ Despite this, the routine clinical adoption of genotyping for warfarin dosing in Qatar is not widespread. Our pilot comprehensively assesses the potential clinical utility of PGx‐guided dosing as a foundational step for broader PGx clinical implementation.

As of February 2022, all patients newly starting warfarin following heart valve replacement surgery at HMC have undergone genotyping, with the goal of improving warfarin dosing and preventing adverse consequences associated with inappropriate dosing. Following agreement to take part, a buccal swab of eligible participants is used for genotyping and analyzed on the POC platform. Genotype results are used readily together with the patient's clinical data, including patient age, height, weight, self‐reported race/ethnicity, smoking status, amiodarone use, and the presence of a recent normal INR as available in the EHR, to provide warfarin dose recommendation. The daily warfarin doses are calculated using a web‐based dose calculator utilizing validated algorithms of the International Warfarin Pharmacogenetics Consortium (IWPC)^30^ and the Gage,^31^ freely available through www.warfarindosing.org.

Genotyping is performed by the ParaDNA POC genotyping platform developed by Laboratory of Government Chemist, with results available within 1 h of receiving the patient's buccal swab. Once results are available, they are integrated into the EHR, and the pharmacist enters the genotype information into the consult note, including genotype‐guided dose recommendations. This information is accessible in time to guide the initial warfarin dose within 24 h of post‐surgery. The clinical pharmacist within the team provides daily assessments of patients and refines dose recommendations based on the INR response to previous doses. These adjustments are documented in the patient's EHR during the initial 5–7 days of warfarin therapy or until the patient is discharged. The prescribing physician receives an alert of each new patient with a warfarin PGx‐guided recommendation through an alert system connected to the patient's EHR.

Eligible patients are mandated to provide written informed consent for using their data and to provide samples for research. HMC's IRB approved this research.

### Statins_SLCO1B1/ABCG2/CYP2C9 implementation project

Statins, a class of lipid‐lowering drugs that are highly prescribed to lower blood cholesterol levels, have demonstrated the ability to decrease the risk of cardiovascular disease (CVD) by 20%–30%.^32^ Although statins are well‐tolerated by most patients, skeletal muscle myopathy and hepatic dysfunctions have been reported with all statins to varying extents.^33^ Statin‐associated muscle symptoms (SAMS) are experienced by up to 20% of patients receiving statin therapy, and the symptoms range from mild muscle aches to life‐threatening rhabdomyolysis.^34^ In the absence of an absolute biomarker for muscle damage in SAMS, studies have focused on investigating candidate genes that contributed to SAMS. This mainly involves the solute carrier organic anion transporter family member 1B1 (SLCO1B1 gene).^35^ The common variant p.Q141K (c.421C>A, rs2231142) in ABCG2, and CYP2C9*2 (p.R144C; rs1799853) and CYP2C9*3 (p.I359L; rs1057910) are also included.^11^ For simvastatin and atorvastatin, evidence linking myopathy to rs4149056 in SLCO1B1 is of high quality, and this association has been replicated in randomized trials and clinical practice‐based cohorts.^36^, ^37^ The most recent of the CPIC guidelines provided therapeutic recommendations for statins based on SLCO1B1, ABCG2, and CYP2C9. This update stated a high level of evidence for SLCO1B1 (associated with simvastatin, rosuvastatin, pravastatin, pitavastatin, atorvastatin, fluvastatin, and lovastatin), ABCG2‐ (rosuvastatin), and CYP2C9 (fluvastatin)‐associated myopathy.^11^

Accumulating evidence has shown a strong association between genetic variability of the SLCO1B1 gene and SAMS risk.^38^ Carriers of SLCO1B1 LOF alleles have about 1.3–5 times higher odds of SAMS depending on statin type and dosage.^39^ In Qatar, using preemptive genotyping for statin‐associated clinically actionable variants could have a great potential in driving personalized drug prescription and improving statin personalized therapy. In addition to being among the most common medications prescribed in Qatar, increased risk of SAMS could be predicted in 32.4% of Qataris who are reported to harbor poor/decreased function of clinically‐actionable SLCO1B1 diplotypes.^5^

Leveraging genomic data from thousands of Qatari individuals, QPGx‐CARES aims to utilize this data in clinical applications such as statin personalized therapy, which could show the potential clinical utility and cost effectiveness of sequencing a large population. The proposed project is a pilot study to assess the feasibility of integrating PGx test results and recommendations into routine clinical decision‐making for patients being considered for initiating the statin lipid‐lowering therapy. A key objective of this pilot is to establish a pipeline that facilitates the use of the generated PGx results in patient clinical care workflows, while implementing clinically validated PGx‐guided statin therapy as a partially preemptive approach.

In this project, QBB participants program who are identified as high‐risk for atherosclerotic cardiovascular disease (ASCVD) according to the American College of Cardiology (ACC) and American Heart Association (AHA) guidelines, will be prescribed statin for the primary prevention of CVD. Eligible participants meeting this criterion will be directed to the medical clinic at HMC hospital. There, prescriptions tailored to their specific needs will be provided based on their medical assessments and genetic data to ensure personalized care (Figure 4c).

The utilization of a partially preemptive testing design will act as a proof of concept for potential future expansion toward a fully preemptive approach. While our immediate focus is on providing pharmacogenomics (PGx)‐guided prescriptions for statin medications, this model offers the opportunity to leverage discrete genetic data for a wider range of gene–drug interactions that may influence prescribing decisions beyond the scope of current medications. By laying the groundwork with this approach, we can explore how preemptive genetic testing can be applied more broadly to enhance personalized medicine across various medical interventions and conditions.

## PGx CURRENT RESEARCH DIRECTIONS AND REFLECTION ON PGx IMPLEMENTATION

Qatar has a unique population with a diverse genetic makeup due to its history of migration and intermarriage.^40^ Various stakeholders in the country have been actively engaged in PGx research and initiatives aimed at advancing personalized medicine and the implementation of PGx testing in clinical settings. PGx research conducted in Qatar focused on the exploration of Qatari‐specific genetic variations related to CVS medication response, mainly warfarin and clopidogrel.^27^, ^41^ Further investigations have been conducted to explore various aspects of warfarin genomics, including the genetic influence on warfarin‐rifampin interaction, INR normalization in periprocedural management, and the economic benefits of genotype‐guided interruption in warfarin pre‐procedural management. Such studies pave the way toward CVS PGx clinical implementation for the local population.^42^, ^43^, ^44^

The QGP has undertaken a substantial sequencing initiative, aiming to generate whole‐genome sequence analyses for more than 30,000 genomes, intending to establish a comprehensive genetic repository tailored specifically to the Qatari population.^45^ Within the framework of QPGx‐CARES, a QGP initiative, researchers have access to valuable resources essential for conducting PGx studies. These resources encompass DNA samples, genetic profiles, and comprehensive health information. This rich dataset serves as a foundation to facilitate in‐depth investigations in the field of PGx by providing researchers with valuable genetic variations specific to the local population. Understanding these genetic variations is crucial for tailoring PGx testing and interventions in the state of Qatar.

To further foster the ability for the clinical implementation of PGx testing, the current PGx implementation research in Qatar focuses on identifying and addressing barriers to implementing PGx testing in clinical settings. This could involve studying factors such as cost effectiveness, ethical considerations, healthcare professionals' education, and integrating genetic information into EHR. In line with the implementation pilots, validation studies are also conducted to evaluate the effectiveness and clinical utility of PGx testing. These studies help establish nationwide recommendations for implementing genetic testing in routine clinical practice.

Of significance, and parallel to the PGx implementation pilots, a series of simulation‐based pharmacoeconomic analyses are currently underway, led by a collaboration between Qatar University, HMC and QGP, to assess the cost‐effectiveness of the PGx‐guided strategy of therapy in Qatar, evaluating the trade‐off between the non‐protocol cost of genotyping, including the POC, and the clinical and economic benefits to generate, primarily in relation to CVS, cancer, and mental health medications. Cost‐effectiveness models are currently being constructed, relying on the high‐quality published literature, incorporating local allele frequencies, and leveraging pertinent economic data. This approach ensures that the cost‐effectiveness analyses are grounded in relevant and region‐specific information, enhancing the accuracy and applicability of the results within the context of QPGx‐CARES. The hypothesis posits that PGx‐guided therapy may demonstrate cost‐effectiveness in alignment with the objectives of QPGx‐CARES. However thorough evaluations are essential to ascertain the real‐life return on investment and validate the positive clinical utility associated with PGx implementation.

## CHALLENGES, GAPS, AND OPPORTUNITIES FOR PGx CLINICAL IMPLEMENTATION IN QATAR

Supporting the PGx clinical implementation, Qatar is building a robust infrastructure essential for the successful integration of PGx testing into routine clinical care. Qatar hosts specialized laboratory facilities equipped with various genotyping platforms capable of analyzing genetic variations relevant to drug response. These include gene‐panel arrays, targeted genotyping assays, and next‐generation sequencing platforms. Different specialized laboratories throughout the country have restricted quality control measures to ensure the accuracy, reliability, and reproducibility of genetic testing. On the other hand, implementing PGx in real‐world settings can encounter various challenges, particularly in specific regions such as Qatar. Addressing these challenges would require collaborative efforts among healthcare professionals, policymakers, researchers, and the community to integrate pharmacogenetics into clinical practice in Qatar effectively.

One of the significant challenges is the need for more appropriate infrastructure in EHRs to store structured genetic data. Integrating genetic data with diverse formats into the existing health IT system, particularly within Cerner or other systems, requires standardization protocols to ensure compatibility and coherence with other clinical data. Qatar's main health systems, HMC and Primary Health Care Centers, are utilizing Cerner Platform in which integrating genetic information seamlessly into routine clinical practice represent a major challenge for us. Additionally, we found a challenge in ensuring seamless interoperability between QBB's data and healthcare facilities owing to the usage of different EHR platforms. In this regard, we are establishing a protocol as part QPGx‐CARES framework to ensure seamless interoperability between genetic databases and clinical decision support systems such as Cerner. The main feature of our workflow is to allow these systems to communicate effectively and exchange information without compromising accuracy and security. Additionally, as a part of the IT solution, we are working to developing algorithms and decision support tools (CDS) that effectively utilize genetic information to guide treatment decisions without overwhelming clinicians.

Addressing ethical and cultural concerns related to genetic testing, data sharing, and informed consent is challenging. Cultural beliefs and privacy concerns might influence patients' acceptance and participation in PGx programs. In this term, through QPGx‐CARES, we are working to provide a regulatory framework to address ethical and legal considerations that encompass patient consent, data privacy, and cultural perceptions regarding genetic testing and personalized medicine, which could impact the acceptance and uptake of PGx in Qatar. Establishing a comprehensive database of genetic information and clinical data, required for effective PGx implementation, might pose challenges regarding collection, storage, and accessibility of this information. We found that translating the vast amount of genetic and clinical data collected by QBB into clinically relevant information for individual patient care is challenging and requires sophisticated analytical tools and methodologies to interpret and apply the data effectively. In terms of data collection, challenges are faced in terms of the integration of different formats and sources of genetic and clinical data. Through our pilots, we are working to develop standardized protocols and formats for data collection, and to ensure that the database is accessible and user‐friendly for healthcare providers.

One of the major challenges we faced during our implementation pilots was the limited awareness and education among healthcare professionals regarding PGx and its clinical applications. In this regard, we provide adequate training for healthcare staff involved in the pilot implementation to comprehend and apply the genetic information in clinical decision‐making. In Qatar, various education program is offered by Qatar University to students and healthcare providers regarding PGx principles and their application in clinical decision‐making. Challenges and solutions for implementing PGx testing in routine clinical care in Qatar are summarized in Table 2.

**TABLE 2:** Challenges and solutions for implementing PGx testing in routine clinical care in Qatar.

Challenges	Description	Solutions
Data management and integration	Integrating genetic data into existing healthcare systems and electronic health records	Developing robust data management protocols and systems that comply with existing IT infrastructure
Data regulation and ethical consideration	Addressing ethical dilemmas related to genetic testing, informed consent, and data sharing	Formulation of regulatory and policy frameworks
Data collection and PGx database	Different formats and sources of genetic and clinical data pose challenges in standardization and integration	Develop standardized protocols and formats for data collection. Utilize interoperable systems and tools to integrate genetic and clinical data
Healthcare professional training	Limited awareness and education: among healthcare professionals regarding PGx use in clinics	Training for healthcare providers to be knowledgeable and confident in interpreting and utilizing PGx information

## CONCLUSION AND FUTURE DIRECTIONS

From a procedural standpoint, we have found that the clinical implementation of PGx‐guided prescriptions of clopidogrel and warfarin is feasible and well‐accepted by healthcare providers. There is a possibility of exporting the components of the PGx‐guided prescribing service, including the genotyping procedure and the clinical decision support tools, to other clinical settings. A future aim, therefore, is to expand the POC PGx testing service to include examination of the effects of genotype‐guided therapy of antiplatelets and anticoagulants in diverse patient populations with different medical conditions such as stroke, peripheral artery disease, venous thrombosis, and atrial fibrillation. QGPx‐CARES's future approach for the transitioning from single gene testing to preemptive panels starts with utilizing a partial preemptive approach in response to statin prescriptions using a multiple‐gene panel. The pilot implementation of the PGx‐guided statin therapy will be used as a proof‐of‐concept project to utilize preemptive PGx testing using multi‐gene panel that covers widely clinically validated gene‐drug pairs with actionable dosing guidelines. The results of the pilot implementation will provide insights into the challenges that may arise during the full implementation of PGx services, such as the need for additional infrastructure, staff training, and patient education. This information can then be used to develop a plan and framework for the successful implementation of PGx services at a national level. Evaluation of the QPGx‐CARES program for PGx testing implementation will provide continuous quality improvement; whereby, by evaluating the effectiveness of the program, any areas of improvement can be identified and addressed, such as improved communication between stakeholders, better staff training, and improved data collection and storage. This will ensure that the program runs smoothly and efficiently. The clinical utility of PGx implementation will be evaluated through observational studies. Such studies can also assess the cost‐effectiveness of PGx testing, which will help adopt this approach. The QPGx‐CARES implementation projects provide the foundation for individualizing medication based on genetic makeup and resolving identified barriers in an actual implementation setting from the perspective of all relevant stakeholders. The prospective outcome of the QPGx‐CARES initiative can provide a better understanding of how PGx data can be used in the real world to help individualize medication and optimize patient outcomes.

## CONFLICT OF INTEREST STATEMENT

The authors declared no competing interests for this work.

## FUNDING INFORMATION

The publication of this article was funded by Qatar National Library.
